# Economics Teachers’ Emotional Intelligence and Self‐Efficacy: A Moderated Mediation Model Using PLS‐SEM

**DOI:** 10.1002/brb3.70832

**Published:** 2025-09-02

**Authors:** Francis Arthur, Regina Okyere‐Dankwa, Dominic Owusu, Sharon Abam Nortey

**Affiliations:** ^1^ Department of Business and Social Sciences Education, Faculty of Humanities and Social Sciences Education University of Cape Coast Cape Coast Ghana; ^2^ OLA College of Education Cape Coast Ghana

**Keywords:** creativity‐nurturing behavior, Economics teachers, emotional intelligence, metacognitive awareness, moderated mediation, teaching self‐efficacy

## Abstract

**Purpose:**

Emotional intelligence (EI) plays a crucial role in shaping teachers' confidence in the classroom. As educators face increasing challenges in today's dynamic educational environment, understanding how EI influences teaching self‐efficacy is essential for enhancing instructional quality. This study investigates the influence of EI on the teachers' self‐efficacy (TSE) of Senior High School (SHS) Economics teachers in Ghana. It further examines the mediating role of creativity‐nurturing behavior (CNB) and the moderating role of metacognitive awareness (MA) in this relationship.

**Method:**

A descriptive cross‐sectional survey design was employed, involving a census of 180 SHS Economics teachers from the Kumasi Metropolis of Ghana. Data were collected using adapted scales for EI, CNB, MA, and TSE. Partial least squares structural equation modeling (PLS‐SEM) was used to analyze the data and test the moderated mediation model.

**Finding:**

The findings revealed that EI significantly influenced CNB and TSE. Additionally, the study identified that CNB had a significant positive effect on TSE. CNB partially mediated the positive relationship between EI and TSE. MA negatively moderated the relationship between EI and CNB. Again, MA influenced the indirect effect of EI on TSE through CNB.

**Conclusion:**

EI is vital for enhancing Economics teachers' confidence in their instructional abilities. The study underscores the importance of fostering EI, CNB, and MA through professional development programs to improve educational outcomes.

## Graphical abstract

Emotional intelligence significantly enhances Economics teachers’ self‐efficacy through creativity‐nurturing behavior. This indirect effect is partially mediated by creativity‐nurturing behavior and is stronger at lower levels of metacognitive awareness, which negatively moderates the link between emotional intelligence and creativity‐nurturing behavior.

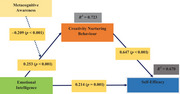

## 1 Introduction

The “United Nations’ Sustainable Development Goal (SDG) 4” emphasizes ensuring “inclusive and equitable quality education” and promoting “lifelong learning opportunities for all” (Boeren 2019). Achieving this goal requires a deliberate focus on empowering teachers to adapt and innovate in response to the dynamic needs of students and society (Rajaram 2023). Teachers’ self‐efficacy—their belief in their ability to facilitate meaningful learning—remains a critical determinant of educational quality (Alibakhshi et al. 2021; Yoon and Goddard 2023). Research indicates that fostering self‐efficacy is integral to enhancing teacher effectiveness, improving student outcomes, and achieving sustainable educational transformation (L. Li and Liu 2022). In the context of Ghana, where education plays a pivotal role in national development, addressing the factors that influence teachers’ self‐efficacy is not just timely but essential for driving economic and social progress.

Despite its importance, teacher self‐efficacy is often challenged by factors such as limited resources, large class sizes, and diverse student needs (Cook and Ogden 2022; Scarparolo and Subban 2021; Weissenfels et al. 2021). Economics teachers, in particular, face the dual responsibility of fostering subject‐specific competencies while also nurturing creativity and critical thinking, both of which are vital for preparing students for the complexities of the 21st‐century economy (Vincent‐Lancrin 2023). These challenges necessitate a deeper understanding of how personal and professional competencies, such as emotional intelligence (EI) and metacognitive awareness (MA), can support teachers in sustaining high levels of self‐efficacy. Addressing these gaps is crucial for equipping Economics teachers with the skills needed to inspire innovation and creativity among students, thereby contributing to Ghana's development agenda.

EI, defined as the “ability to recognize, understand, and manage one's emotions and those of others,” has emerged as a transformative competency for teachers (Gómez‐Leal et al. 2022). By enabling teachers to navigate emotional and social complexities, EI enhances their capacity to create supportive learning environments, manage stress, and build strong teacher–student relationships (X. Wang 2023). However, the extent to which EI directly influences self‐efficacy in teaching remains underexplored, particularly within the Ghanaian educational context. This study seeks to address this gap by examining the role of EI in fostering teaching self‐efficacy among Ghanaian Economics teachers.

In addition to EI, MA—the ability to think about and regulate one's learning processes—has been recognized as a critical factor influencing teaching efficacy (Yidana 2023). MA enables teachers to reflect on their instructional practices, adapt strategies to diverse learning needs, and foster creativity among students (Cheng and Chan 2021). However, the interplay between MA and EI in shaping teaching self‐efficacy has not been sufficiently investigated. This study hypothesizes that MA may moderate the relationship between EI and self‐efficacy, further enhancing our understanding of how these competencies interact to influence teacher performance.

EI and creativity‐nurturing behavior (CNB) have been widely examined in educational research, with findings suggesting that emotionally intelligent teachers foster creative learning environments (Carmeli et al. 2014; Ivcevic et al. 2007). While studies confirm the mediating roles of generosity and vigor in the EI–CNB nexus (Carmeli et al. 2014), little is known about this relationship in the specific context of Economics teachers. Additionally, the interplay between EI and teachers' self‐efficacy (TSE) has been well‐documented, with evidence showing that self‐efficacy mediates the EI–teacher effectiveness relationship (Anwar et al. 2021; L. Wang 2022). However, the indirect role of CNB in this framework remains largely unexplored.

Furthermore, while prior research establishes CNB as a significant predictor of TSE (Ozkal 2014; Xiong et al. 2020), studies have not examined whether CNB acts as a linking mechanism between EI and TSE. This omission limits our understanding of how EI shapes self‐efficacy through creativity‐nurturing practices. Additionally, inconsistencies exist regarding the direct influence of EI on creative behaviors—while some studies suggest a positive relationship (Carmeli et al. 2014), others argue that EI alone is insufficient to predict creativity without mediators such as emotional creativity (Ivcevic et al. 2007). Moreover, research on MA and its influence on CNB is still emerging. Although Tok (2022) found that MA significantly predicts different creative thinking domains, the role of MA in shaping teachers’ CNBs remains underexplored, particularly among Economics teachers. Likewise, while Alkan (2020) and Yidana (2023) established that MA influences TSE, how MA interacts with CNB to enhance TSE has not been thoroughly investigated.

This study contributes to the global discourse on teacher empowerment and educational quality by situating its inquiry within the Ghanaian context. Employing a moderated mediation model analyzed through “partial least squares structural equation modeling (PLS‐SEM),” the research aims to unpack the complex interrelationships among EI, MA, CNB, and teaching self‐efficacy. Additionally, the study advances the understanding of how EI and MA interact to influence teachers' self‐efficacy (TSE) through CNB.

## 2 Theoretical Foundation

The theoretical foundation of this study is built upon Bandura's (1986) “social cognitive theory (SCT),” Goleman's (1995) “emotional intelligence theory (EIT),” and T. Amabile's (2011) “componential theory of creativity.” Individually and collectively, these theories provide a comprehensive framework for understanding how “emotional intelligence (EI)” influences teachers’ instructional confidence through “creativity‐nurturing behavior (CNB)” and the moderating role of “metacognitive awareness (MA).” SCT emphasizes the interplay between personal factors, behaviors, and environmental influences, with self‐efficacy as a core component. In the context of teaching, self‐efficacy reflects an educator's belief in their ability to effectively facilitate learning and manage classroom challenges. Bandura (1986) posits that self‐efficacy evolves through mastery experiences, vicarious learning, and emotional regulation (J. Li et al. 2025; Usher et al. 2023). This theoretical perspective aligns with the study's objective of examining how EI contributes to teachers’ confidence in fostering creativity and navigating instructional complexities.

Goleman's (1995) “emotional intelligence theory” complements SCT by emphasizing the role of emotional awareness, regulation, and social skills in professional effectiveness. EI is critical in equipping teachers with the ability to manage interpersonal relationships, cope with stress, and navigate the social dynamics of the classroom (Goleman 1995; Petrides 2010). Given that effective teaching requires both cognitive and emotional competencies, EIT provides a valuable lens through which the study examines the impact of EI on “teachers’ creativity‐nurturing behavior and self‐efficacy.” By linking EI to key instructional outcomes, the study contributes to the growing body of research demonstrating that emotionally intelligent teachers are more adept at fostering student engagement and facilitating a positive learning environment.

T. Amabile's (2011) “componential theory of creativity” further strengthens the theoretical foundation by explaining how creativity is driven by domain‐relevant skills, intrinsic motivation, and an enabling environment. In teaching, CNB is essential for fostering student innovation and engagement (T. M. Amabile and Mueller 2024). This study adopts the “componential theory of creativity” to support the mediating role of CNB in the relationship between EI and “teachers' self‐efficacy (TSE),” demonstrating that teachers who nurture creativity not only enhance student learning experiences but also reinforce their own instructional confidence. The theory underscores that fostering creativity is a dynamic process influenced by both individual and environmental factors (Mirza et al. 2024), aligning with the study's exploration of how EI shapes CNB and, in turn, TSE.

Collectively, these theories provide a robust foundation for investigating the moderated mediation model in this study. SCT highlights self‐efficacy as a central mechanism in effective teaching, EIT elucidates how emotional competencies contribute to instructional confidence, and the componential theory of creativity explains the importance of CNB in translating EI into greater teaching self‐efficacy.

## 3 Literature Review and Hypotheses Development

### 3.1 EI and CNB

EI refers to an “individual's ability to recognize, understand, and manage their emotions and those of others, which fosters positive social interactions and decision‐making” (Watanabe et al. 2024). CNB involves actions and attitudes that support, encourage, and enhance creative thinking and problem‐solving in oneself and others (Apak et al. 2021). EI has been widely recognized as a key predictor of creativity in educational contexts (Pashazadeh and Alavinia 2019). Teachers with higher EI are better able to understand and manage their emotions, as well as those of their students, which can foster an environment conducive to creativity (Mayer and Salovey 1997; Su et al. 2024). Prior research has identified a significant link between EI and creativity (Carmeli et al. 2014; Tu et al. 2020; Xu et al. 2019). However, Ebrahimi et al. (2018) reported no significant impact of EI on creativity. In the context of Economics teachers, EI could positively influence their ability to nurture creativity in their teaching practices, encouraging innovative and adaptive teaching strategies that enhance student engagement and learning outcomes (Su et al. 2022; Su et al. 2024). Therefore, it is hypothesized as follows:

*H1: Emotional intelligence (EI) positively influences creativity‐nurturing behavior (CNB)*.

### 3.2 CNB and Self‐Efficacy

CNB refers to teachers’ actions that foster creativity in students, such as encouraging critical thinking, open‐mindedness, and innovative problem‐solving (Apak et al. 2021; Mahama et al. 2023). Engaging in CNB enhances teachers' self‐efficacy by increasing their confidence in their ability to create dynamic and effective learning environments. Creativity is considered a vital component of effective teaching and teacher self‐efficacy (Hayati et al. 2023). Teachers who engage in CNBs, such as fostering open‐mindedness, creating an innovative classroom environment, and promoting critical thinking, are likely to feel more confident in their teaching abilities (Xiong et al. 2020). This increased sense of competence is aligned with higher teacher self‐efficacy (Bandura 1997). Teachers who nurture creativity often incorporate innovative techniques, approaches, and resources to enhance the learning experience and make it more engaging (Ma 2022). For example, Xiong et al. (2020) and Ozkal (2014) found a positive link between CNB and teaching self‐efficacy. Therefore, CNB is hypothesized to have a positive influence on TSE, as teachers who nurture creativity in their classrooms are more likely to believe in their teaching capabilities.

*H2: “Creativity‐nurturing behavior (CNB) positively influences teachers' self‐efficacy (TSE)*.”

### 3.3 EI and Self‐Efficacy

EI refers to an “individual's ability to recognize, understand, manage, and regulate their own emotions and those of others to facilitate thinking and behavior” (Rodrigues and Junça Silva 2024; Yousaf et al. 2024). Teachers' self‐efficacy (TSE) is a teacher's belief in their ability to effectively plan, instruct, and manage classroom activities to enhance student learning outcomes (Calkins et al. 2024; Yidana & Arthur, 2023). EI enhances teachers’ ability to manage emotions, build positive relationships, and handle classroom challenges, which in turn boosts their confidence in their teaching abilities, known as teachers' self‐efficacy (TSE) (Lu and Ishak 2022). Empirical studies suggest that teachers with high EI are more likely to feel competent in engaging students, maintaining discipline, and delivering effective instruction (Ye et al. 2024). Research consistently supports the link between EI and teacher self‐efficacy (Chen et al. 2024; Kyriazopoulou et al. 2025; Ye et al. 2024; Zhi et al. 2024). Teachers with high EI tend to have better interpersonal relationships with their students, manage classroom dynamics more effectively, and demonstrate more confidence in their teaching abilities (Schutte et al. 2001; Valente and Lourenço 2020; Wu et al. 2019). Studies (e.g., Anwar et al. 2021; Kostić‐Bobanović 2020; Saidi 2020; Valente et al. 2020) have shown a positive relationship between EI and TSE. However, Kyriazopoulou et al. (2025) and Udayar et al. (2020) revealed that there was a negative connection between EI and TSE. In the context of Economics teaching, EI is expected to enhance teachers' self‐efficacy, as it helps them navigate the challenges of the classroom and engage students in meaningful ways. Thus, it is hypothesized that EI directly influences TSE positively.

*H3: Emotional intelligence (EI) positively influences teachers' self‐efficacy (TSE)*.

### 3.4 Mediating Role of CNB

The positive effects of EI on TSE are likely to be mediated by CNB. Teachers who possess high EI are better equipped to create a supportive and creative learning environment, which, in turn, enhances their belief in their teaching abilities (Su et al. 2022). This mediation model suggests that EI leads to higher CNB, which subsequently boosts TSE. Therefore, CNB is expected to mediate the relationship between EI and TSE, with teachers’ CNBs serving as a conduit through which EI enhances their self‐efficacy.

*H4: Creativity‐nurturing behavior (CNB) will mediate the positive relationship between emotional intelligence (EI) and teachers' self‐efficacy (TSE)*.

### 3.5 Metacognitive Awareness

MA involves teachers' ability to reflect on and regulate their cognitive processes in relation to teaching and learning (Akcaoğlu et al. 2023; Huang et al. 2022; Mbato and Triprihatmini 2022). Teachers with high MA are more likely to engage in strategic thinking, evaluate their teaching practices, and adapt them to foster creativity in the classroom (Hiver et al. 2021). Also, Huang et al. (2021), Maor et al. (2023), and Tok (2022) revealed a significant connection between teachers’ metacognition and creativity. In the context of Economics teaching, it is expected that higher MA will promote behaviors that nurture creativity, as teachers will be more reflective and open to innovative approaches in their teaching. Therefore, MA is hypothesized to positively influence CNB.

*H5: “Metacognitive awareness (MA) positively influences creativity‐nurturing behavior (CNB)*.*”*

Although EI and MA are both important for teaching self‐efficacy, their interaction may not always lead to a simple additive effect. When teachers possess high levels of both EI and MA, they may be more focused on emotional regulation and reflective practice, which could lead to a less intense emphasis on creativity in their teaching approaches. The negative moderation hypothesis suggests that the combined effect of EI and MA might decrease the intensity of the relationship between EI and CNB. This moderation effect can be explained through “cognitive load theory (CLT),” which posits that individuals have a limited cognitive capacity for processing information (Sweller 2011). Teachers with high levels of both EI and MA may experience increased cognitive demands due to their simultaneous focus on emotional regulation and reflective practice. As a result, they might allocate more cognitive resources to structured, well‐organized teaching approaches rather than fostering CNBs. This aligns with CLT's assertion that when cognitive load is high, individuals may prioritize efficiency and structure over exploratory or creative processes (Chen et al. 2023; Krieglstein et al. 2023). Hence, it is hypothesized that the interaction between EI and MA negatively moderates the relationship between EI and CNB.

*H6: “The interaction between emotional intelligence (EI) and metacognitive awareness (MA) negatively moderates the relationship between emotional intelligence (EI) and creativity‐nurturing behavior (CNB)*.”

Given the moderating role of MA, it is expected that the indirect relationship between EI and TSE, mediated by CNB, will vary depending on the level of MA. Empirical studies (e.g., Alkan 2020; Yidana 2023; Yildiz and Akdag 2017) found that teachers’ MA positively influenced their teaching self‐efficacy. Teachers with higher MA may be more reflective about the creative processes in their classrooms, thereby enhancing the effect of EI on CNB and, ultimately, TSE. Conversely, teachers with lower MA may not fully capitalize on the creative potential fostered by EI, resulting in a weaker indirect effect. Therefore, the indirect effect of EI on TSE through CNB is hypothesized to be moderated by MA, with higher levels of MA strengthening the relationship.

*H7: The indirect effect of emotional intelligence (EI) on teachers' self‐efficacy (TSE) through creativity‐nurturing behavior (CNB) is moderated by metacognitive awareness (MA)*.

### 3.6 Conceptual Model

**FIGURE 1:**
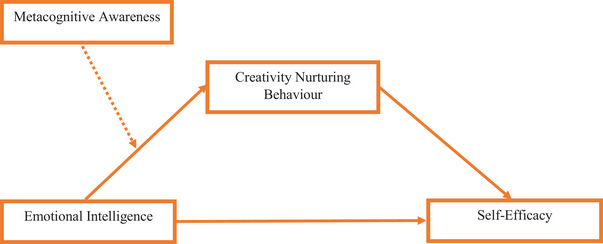
Moderated mediation model. *Source*: Authors’ construct.

Figure 1 illustrates a moderated mediation model investigating the relationships among “emotional intelligence, creativity‐nurturing behavior, metacognitive awareness, and self‐efficacy.” EI serves as a predictor, influencing “creativity‐nurturing behavior,” which mediates its influence on “teaching self‐efficacy.” Additionally, “metacognitive awareness” moderates the relationship between “emotional intelligence and creativity‐nurturing behavior,” highlighting how variations in metacognitive capacity influence this pathway.

## 4 Materials and Methods

### 4.1 Study Design and Sample

The study adopted a descriptive cross‐sectional survey design to examine the relationship between Economics teachers’ EI and teaching self‐efficacy. The target population comprised all Economics teachers in “Senior High Schools (SHSs)” within the “Kumasi Metropolis of Ghana.” According to the “Ghana Education Service” (GES 2024), the Metropolis had 67 SHSs, collectively employing 335 “Economics teachers.” Using simple random sampling, 30 out of the 67 schools were selected for the study. The “census method” was employed to include all 185 Economics teachers from the 30 selected schools to avoid sampling bias (Stratton 2023). Also, the number of respondents was deemed appropriate because the “G*Power sample size” calculation recommended a sample of 119 for a model with three predictors (see Appendix A).

### 4.2 Measures

The study utilized well‐validated instruments to capture key constructs. EI was measured using an adapted “emotional intelligence scale (16 items)” by Law et al. (2004). Teachers’ MA was assessed with the “Metacognitive Awareness Inventory for Teachers (MAIT)” (24 items) developed by Balcikanli (2011), while “creativity‐nurturing behavior (15 items)” was evaluated through a standardized tool designed by Sharma and Sharma (2018). Teaching self‐efficacy was measured using the “teacher sense of efficacy scale (TSES)” (24 items), developed by Tschannen‐Moran and Woolfolk Hoy (2001). Also, all the items were measured on a 7‐point Likert scale ranging from “*strongly disagree* (1)” to “*strongly agree* (7).” Each instrument was chosen for its reliability and established psychometric properties in educational research.

### 4.3 Data Collection Procedure

Four research assistants were engaged and trained extensively on the study's objectives, the administration of the questionnaire, and ethical considerations, including confidentiality and voluntary participation. Each assistant was assigned to a specific school within the selected sample. They distributed the questionnaires to Economics teachers and provided 50–60 min for completion. To ensure data accuracy and completeness, the assistants immediately reviewed the filled questionnaires upon collection. Out of the 185 questionnaires distributed, 180 were retrieved, representing a return rate of 97.3%.

### 4.4 Data Analysis Strategy

The data were analyzed using “partial least squares structural equation modeling (PLS‐SEM)” through “SmartPLS 4 software” (Ringle et al. 2022). This analytical approach was chosen due to its suitability for complex models, particularly those involving mediation and moderation effects. The “PLS‐SEM” approach facilitated the evaluation of the relationships between “emotional intelligence, creativity‐nurturing behavior, metacognitive awareness, and teaching self‐efficacy” while testing the moderating and mediating effects within the proposed model.

### 4.5 Ethical Considerations

Ethical considerations were rigorously observed in this study to ensure the rights and well‐being of all respondents. Confidentiality and anonymity were strictly maintained by assigning unique identifiers to the data, ensuring that no personal information could be traced back to individual participants. Respondents were fully informed about the purpose, procedures, and potential implications of the study through a detailed “informed consent form,” which they were required to read and sign before participation. Participation was entirely voluntary, and respondents were assured of their right to withdraw at any stage without any consequences. These measures aligned with established ethical guidelines for research involving human subjects. Ethical approval was obtained from the “Institutional Review Board of the anonymized.”

## 5 Results

### 5.1 Multivariate Normality

The assessment of “multivariate normality,” using “Mardia's test,” revealed significant skewness (*b* = 9.039, *z* = 180.783, *p* < 0.05) and kurtosis (*b* = 35.415, *z* = 9.024, *p* < 0.05) (see Appendix B), indicating a deviation from “multivariate normality” (Hair, Risher et al. 2019). Due to this violation, “PLS‐SEM” was employed, as it is a robust technique that does not require data to meet the “assumption of multivariate normality” (J. Hair et al. 2022). This makes “PLS‐SEM” suitable for the analysis of the current dataset.

### 5.2 Common Method Bias

Full collinearity “variance inflation factors (VIFs)” were assessed, with all values below 3.3, confirming the absence of significant “collinearity” concerns (Kock 2015). Harman's single‐factor test further indicated that the largest single factor accounted for less than 50% of the total variance, demonstrating that “common method bias” is not a significant issue (Podsakoff et al. 2003).

### 5.3 Measurement Model

The “measurement model analysis” revealed that poorly loading items were deleted to enhance the reliability and validity of the constructs. The results in Table 1 revealed that the “item loadings” ranged from 0.508 to 0.827 (J. Hair et al. 2022). The “average variance extracted (AVE)” values of the constructs were above the recommended threshold of 0.50 (see Table 1) (J. Hair et al. 2022), and also the respective “composite reliability (CR)” values exceeded 0.70, indicating sufficient “convergent validity” (Hair, Black et al. 2019). Further deletion of items was explored but did not significantly improve the “AVE values,” affirming the adequacy of the retained items. The constructs demonstrated robust “internal consistency,” as evidenced by high “Cronbach's alpha (CA) and CR values,” ensuring the reliability of the “measurement model.”

**TABLE 1:** Measurement model.

Constructs	No. of items	*λ* range	CA (*α*)	rho_A	CR	AVE
Creativity‐nurturing behavior (CNB)	15	0.565–0.795	0.925	0.932	0.935	0.592
Emotional intelligence (EI)	16	0.558–0.775	0.917	0.921	0.928	0.548
Metacognitive awareness (MA)	24	0.508–0.753	0.932	0.937	0.939	0.596
Teachers' self‐efficacy (TSE)	18	0.621–0.827	0.942	0.944	0.948	0.506

*Note*: λ = indicator loading.

### 5.4 Discriminant Validity

Discriminant validity was assessed using the “Heterotrait‐Monotrait (HTMT) ratio,” as shown in Table 2. The HTMT values for all construct pairs were below the threshold of 0.90, demonstrating that the constructs are empirically distinct from one another. Specifically, the “HTMT ratios” ranged from 0.691 to 0.885, indicating acceptable levels of “discriminant validity” and supporting the “uniqueness of each construct” in the model (Henseler et al. 2015). These results confirm that the constructs measure conceptually distinct phenomena, thereby enhancing the rigor and validity of the structural model.

**TABLE 2:** HTMT ratio.

Construct	CNB	EI	MA	TSE
CNB				
EI	0.712			
MA	0.816	0.730		
TSE	0.836	0.691	0.885	

### 5.5 Evaluation of Structural Model

**FIGURE 2:**
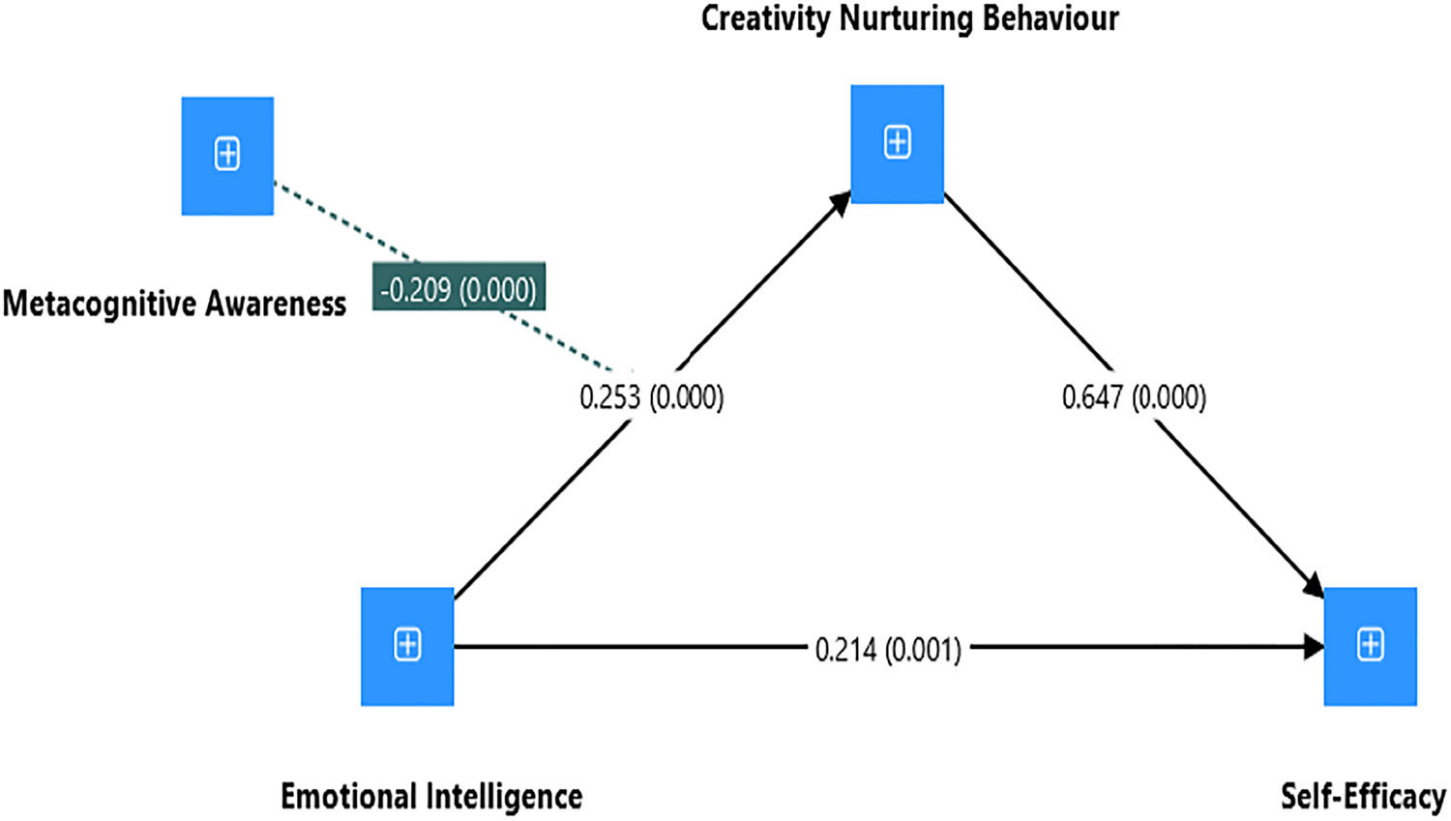
Structural model of the moderated mediation analysis (SmartPLS output).

In evaluating the structural model, a bootstrapping procedure with 10,000 resamples was conducted after ensuring that there were no issues with “multicollinearity,” as indicated by the “variance inflation factors (VIFs)” all being below the threshold (VIFs ranged from 1.389 to 2.248) (J. Hair et al. 2022). Figure 2 shows the moderated mediation model generated using SmartPLS 4 software. For instance, the path from CNB to TSE (*β* = 0.647, *t* = 8.401, *p* < 0.001) demonstrated a strong, significant positive relationship (see Table 3). The path from EI to CNB (*β* = 0.253, *t* = 3.604, *p* < 0.001) also showed a significant positive effect. The MA to CNB relationship (*β* = 0.430, *t* = 4.694, *p* < 0.001) was similarly significant. Additionally, the specific indirect effects show that the indirect effect of MA on TSE through CNB is significant (*β* = 0.278, *t* = 3.953, *p* < 0.001). Moreover, the interaction between MA and EI in predicting TSE through CNB reveals a significant moderated indirect effect (*β* = −0.135, *t* = 3.410, *p* = 0.001) (see Table 3).

**TABLE 3:** Structural model.

Relationships	*β*	SD	*T*‐values	*p* values	VIF	2.5%	97.5%	$f^{2}$	$R^{2}$
Direct effect									
EI → CNB	0.253	0.070	3.604	< 0.001	1.905	0.116	0.394	0.130	0.723
CNB → TSE	0.647	0.077	8.401	< 0.001	1.871	0.489	0.782	0.673	0.670
EI → TSE	0.214	0.065	3.262	0.001	1.871	0.086	0.344	0.086	
MA → CNB	0.430	0.092	4.694	< 0.001	2.248	0.239	0.604	0.299	
Moderation									
MA × EI → CNB	−0.209	0.056	3.737	< 0.001	1.389	−0.292	−0.068	0.268	
Moderated mediation									
MA × EI → CNB → TSE	−0.135	0.040	3.410	0.001	—	−0.200	−0.041		
Indirect effect									
MA → CNB → TSE	0.278	0.070	3.953	< 0.001	—	0.142	0.417		

Next, the model's *f* ^2^ values were calculated to assess the effect sizes. The *f* ^2^ values were interpreted based on Cohen's (1988) guideline, where values of 0.02, 0.15, and 0.35 represent small, medium, and large effects, respectively. Notably, the MA to CNB path exhibited a medium effect size (*f* ^2^ = 0.299). This denotes a substantial effect, approaching the threshold for a large effect size, and implies that MA plays a critical role in enhancing CNB. Also, the effect size for the EI to TSE path was small (*f* ^2^ = 0.086). Although comparatively small, this finding still reflects a meaningful contribution of EI to the development of TSE. The MA and EI interaction on CNB showed a significant negative effect (*β* = −0.209), with an *f* ^2^ of 0.268, indicating a medium effect size as well. This suggests a medium effect size, underscoring the meaningful contribution of the interaction term in predicting changes in CNB. Taken together, the effect size results underscore the theoretical and practical relevance of MA and EI in predicting the outcome variables, highlighting their differential impact across the structural model.

Finally, the *R* ^2^ values were examined, with the model explaining 67% of the variance in TSE and 72.3% of the variance in CNB, indicating strong explanatory power for both outcome variables. The *R* ^2^ value for CNB is 0.723, which implies that approximately 72.3% of the variance in CNB is explained by EI, MA, and their interaction term (MA × EI). This high level of explained variance indicates that these predictors collectively provide strong explanatory power for CNB. Among these, MA (*β* = 0.430, *f* ^2^ = 0.299) had the most substantial effect, followed by EI (*β* = 0.253, *f* ^2^ = 0.130), and the interaction term contributed moderately (*f* ^2^ = 0.268), albeit with a negative beta coefficient (*β* = −0.209). These findings suggest that both EI and MA independently and jointly shape the extent to which individuals engage in CNBs.

The *R* ^2^ value for TSE is 0.670, indicating that 67.0% of the variance in TSE is accounted for by CNB and EI. Here, CNB (*β* = 0.647, *f* ^2^ = 0.673) had a strong and direct influence, suggesting it is the most critical predictor of TSE in the model. Although EI also had a direct effect on TSE (*β* = 0.214, *f* ^2^ = 0.086), its contribution was smaller in comparison. This implies that while EI contributes directly to TSE, its impact is more strongly mediated through CNB. These results provide a clear understanding of the key relationships and effect sizes within the model, offering insight into the dynamics of EI, MA, and CNB in shaping TSE.

### 5.6 Moderated Indirect Relationship

The analysis of the moderated indirect relationship between EI, CNB, and TSE reveals a significant indirect effect (*β* = 0.163, *p* < 0.001) (see Table 4). This means that CNB partially mediated the relationship between EI and self‐efficacy. When examining the moderation by MA, the effect of EI on CNB to TSE was stronger at lower levels of MA (−1 SD, *β* = 0.298, *p* < 0.001) but weak and nonsignificant at higher levels of MA (+1 SD, *β* = 0.028, *p* = 0.565). At the mean level of MA, the indirect effect was significant (*β* = 0.163, *p* < 0.001). The index of moderated mediation was significant (*β* = −0.135, CI = −0.200 to −0.041, *p* = 0.001), indicating that MA moderates the indirect relationship between EI and TSE.

**TABLE 4:** Moderated indirect relationship.

Moderated indirect relationship	Direct effect	Indirect effect	Confidence interval low/high	*p* values
EI → CNB → TSE	0.214 (3.262)	0.163 (3.512)	0.077/0.262	< 0.001
Probing moderated indirect relationships				
EI → CNB → TSE conditional on MA at −1 SD (low level)		0.298	0.142/0.425	< 0.001
EI → CNB → TSE conditional on MA at +1 SD (high level)		0.028	−0.054/0.137	0.565
EI → CNB → TSE conditional on MA at mean (mean level)		0.163	0.077/0.262	< 0.001
Index of moderated mediation		−0.135	−0.200/−0.041	0.001

*Note*: Value in bracket is *t*‐statistics/value.

**FIGURE 3:**
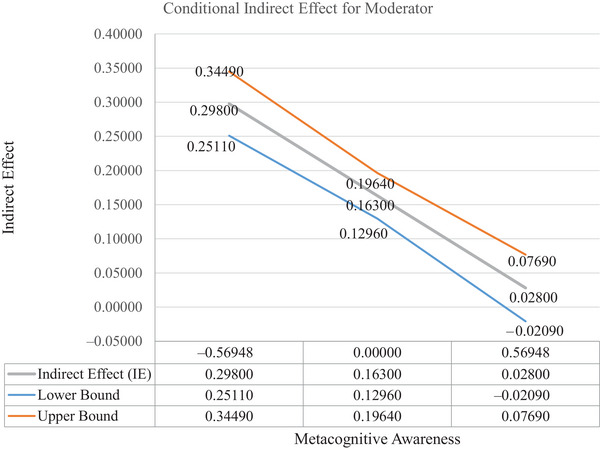
Moderated mediation plot.

Furthermore, the results indicate that the index value of MA for the moderated mediation effect is significant [index (*ω*) = −0.135, SE = 0.040, 95% CI = (−0.200, −0.041)]. The results revealed that at the higher level of MA, the indirect effect of EI on self‐efficacy through CNB (path = 0.028, *t* = 0.576, *p* = 0.565) is lower in comparison to the indirect effect of low MA (path = 0.298, *t* = 4.198, *p* < 0.001) (see Table 4). This shows that with the increase in MA, the indirect effect of EI on TSE through CNB is reduced. Hence, the moderated mediation model is supported. Slope analysis (see Figure 3 and Appendix C) also further supports the results and shows that with an increase in MA, the indirect effect of EI on TSE through CNB is reduced. This suggests that in order to maximize the positive effect of EI on TSE, it is important to maintain a balance between MA and CNB.

## 6 Revised Conceptual Framework

**FIGURE 4:**
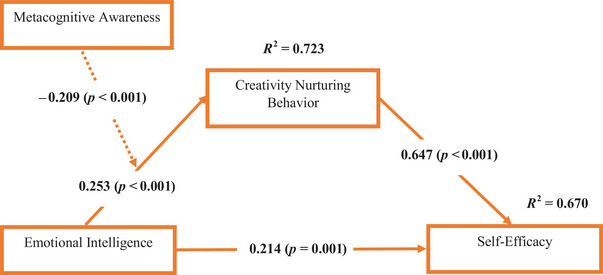
Revised conceptual model. *Source*: Authors’ construct.

The revised conceptual model illustrates the relationships between MA, EI, CNB, and self‐efficacy. It shows that EI positively influences CNB and self‐efficacy, while MA negatively affects CNB, which in turn significantly impacts self‐efficacy (see Figure 4).

## 7 Discussion

### 7.1 EI and CNB

The current study's finding that EI significantly influences teachers' CNB aligns with prior research, which underscores the role of EI in fostering creativity in educational settings (Pashazadeh and Alavinia 2019; Su et al. 2024). EI enables teachers to manage their emotions effectively and create an environment that supports creative thinking and problem‐solving among students (Mayer and Salovey 1997). This confirms the notion that emotionally intelligent teachers can employ strategies that enhance student engagement and innovation (Carmeli et al. 2014; Tu et al. 2020). While some studies have established a strong link between EI and creativity (Xu et al. 2019), conflicting evidence exists, such as the findings of Ebrahimi et al. (2018), which did not establish a significant relationship. However, the present study's findings reinforce the perspective that EI remains a crucial factor in shaping creativity‐supportive teaching behaviors, particularly in the Economics discipline (Su et al. 2022).

This finding may stem from the increasing emphasis on student‐centered pedagogies that require teachers to be adaptable and innovative (Watanabe et al. 2024). Economics teachers with higher EI are likely better at recognizing students' learning needs, adjusting their teaching methods, and fostering an interactive and explorative classroom environment (Apak et al. 2021). The nature of Economics as a subject demands critical thinking and problem‐solving skills, which emotionally intelligent teachers can effectively nurture through creative instructional strategies (Su et al. 2024). Additionally, given the challenges in Ghana's education system, such as limited resources and rigid curricula, emotionally intelligent teachers may compensate by employing CNBs to make learning more engaging and impactful (Su et al. 2022). This underscores the importance of integrating EI development into teacher training programs to enhance instructional effectiveness and student learning outcomes (Pashazadeh and Alavinia 2019).

### 7.2 CNB and Teaching Self‐Efficacy

The current study's finding that CNB positively influences teaching self‐efficacy aligns with existing literature emphasizing the role of creative teaching in fostering educators' confidence in their instructional abilities (Apak et al. 2021; Mahama et al. 2023). Teachers who engage in CNB—such as encouraging critical thinking, open‐mindedness, and problem‐solving—develop a stronger belief in their capacity to create meaningful and dynamic learning experiences (Hayati et al. 2023). This supports the argument that incorporating innovative teaching strategies enhances teachers' sense of competence and self‐efficacy (Xiong et al. 2020). The study's findings also correspond with prior research, which highlights a positive link between creativity‐supportive teaching and higher levels of teaching self‐efficacy (Ozkal 2014; Ma 2022). Bandura's (1997) self‐efficacy theory further reinforces this relationship, suggesting that when teachers successfully implement creativity‐enhancing techniques in their classrooms, they develop greater confidence in their instructional effectiveness.

This positive relationship may stem from the increasing demand for adaptive and student‐centered instructional approaches that require teachers to be innovative in their pedagogical practices (Xiong et al. 2020). Given the complex and analytical nature of Economics, teachers who actively nurture creativity in their classrooms may feel more empowered to engage students in meaningful discussions, problem‐solving tasks, and critical analyses, thereby strengthening their teaching self‐efficacy (Apak et al. 2021). Furthermore, the constrained resources and rigid curricula in Ghanaian education may require teachers to adopt creative instructional strategies to enhance student engagement and understanding (Mahama et al. 2023). This underscores the need for professional development programs that equip teachers with creative pedagogical skills, ultimately reinforcing their confidence in their teaching effectiveness (Hayati et al. 2023).

### 7.3 EI and Teaching Self‐Efficacy

The study's finding that EI positively influences teachers' self‐efficacy (TSE) aligns with existing research that highlights the role of EI in shaping teachers’ confidence in their instructional abilities (Rodrigues and Junça Silva 2024; Yousaf et al. 2024). Teachers with higher EI are better equipped to manage emotions, foster positive classroom relationships, and navigate challenges, which ultimately enhances their self‐efficacy in lesson planning, instruction, and classroom management (Lu and Ishak 2022). This is consistent with prior studies indicating that teachers with strong EI exhibit greater competence in engaging students, regulating classroom behavior, and maintaining an effective learning environment (Ye et al. 2024). The significant relationship between EI and TSE further supports findings that emotionally intelligent teachers experience greater confidence in handling the complexities of teaching, leading to improved instructional effectiveness (Chen et al. 2024; Kyriazopoulou et al. 2025; Zhi et al. 2024). However, contrary findings by Kyriazopoulou et al. (2025) and Udayar et al. (2020) suggest that contextual factors may moderate the relationship between EI and self‐efficacy.

In the Ghanaian context, Economics teachers’ EI may be particularly influential in enhancing their teaching self‐efficacy due to the subject's analytical nature and the need for engaging instructional methods (Calkins et al. 2024; Yidana & Arthur, 2023). Given the often resource‐constrained classrooms, teachers with higher EI may be more adept at managing stress, maintaining student engagement, and fostering a supportive learning environment, which strengthens their belief in their teaching capabilities (Schutte et al. 2001; Valente and Lourenço 2020). Additionally, Economics teachers frequently interact with diverse student needs, requiring strong EI to navigate varying academic abilities and motivations. This suggests that professional development programs aimed at enhancing teachers’ EI could be instrumental in boosting their self‐efficacy, leading to more effective and adaptive instructional practices (Wu et al. 2019; Saidi 2020).

### 7.4 Mediating Role of CNB

The study's finding that CNB partially mediates the positive relationship between EI and teachers' self‐efficacy (TSE) reinforces the idea that emotionally intelligent teachers are more likely to foster creativity in their classrooms, which in turn enhances their confidence in their teaching abilities. Teachers with high EI are adept at managing their emotions and those of their students, creating an environment that encourages critical thinking, open‐mindedness, and problem‐solving. This aligns with previous research indicating that EI contributes to teachers' ability to nurture creativity, ultimately strengthening their belief in their instructional competence (Su et al. 2022). The partial mediation suggests that while EI directly enhances TSE, CNB serves as an additional mechanism through which this influence is reinforced. Teachers who actively engage in CNBs are more likely to feel competent in their ability to implement innovative teaching strategies, leading to higher levels of self‐efficacy.

In the Ghanaian context, where Economics teachers must navigate complex economic theories and real‐world applications, CNB may play a crucial role in bridging the gap between theoretical knowledge and practical understanding. The ability of Economics teachers to integrate creativity into their instructional methods—such as using case studies, simulations, and inquiry‐based learning—could further enhance their confidence in delivering engaging lessons. The partial mediation finding also suggests that other contextual factors, such as institutional support, access to teaching resources, and student engagement levels, may independently contribute to teachers’ self‐efficacy. Therefore, professional development initiatives that simultaneously enhance EI and encourage creativity‐nurturing teaching practices could be instrumental in strengthening teachers’ overall effectiveness in the classroom.

### 7.5 MA and CNB

The study's finding that Economics teachers’ MA positively influences their CNB aligns with existing research highlighting the role of metacognition in fostering creativity in educational settings. Teachers with high MA are more likely to reflect on their teaching strategies, assess their effectiveness, and make adjustments to enhance student engagement and creative thinking (Akcaoğlu et al. 2023; Huang et al. 2022). This finding is consistent with prior studies that have identified a significant link between teachers’ metacognitive skills and their ability to nurture creativity in the classroom (Hiver et al. 2021; Maor et al. 2023). When teachers engage in self‐regulation and strategic thinking, they are better equipped to create learning environments that encourage open‐ended inquiry, problem‐solving, and innovation, ultimately supporting students' creative potential (Tok 2022).

In the context of Ghanaian Economics education, where conceptual understanding and real‐world application are crucial, MA allows teachers to refine their instructional methods to better engage students. Economics teachers who continuously evaluate their pedagogical approaches can implement creative teaching techniques such as scenario‐based learning, debates, and problem‐based instruction, which enhance students’ critical thinking and innovation (Mbato and Triprihatmini 2022). The positive influence of MA on CNB also suggests that professional development programs emphasizing metacognitive skills could further empower teachers to nurture creativity effectively. Encouraging teachers to consciously reflect on their teaching methods and student responses may contribute to more adaptive, engaging, and innovative classroom practices (Huang et al. 2021).

### 7.6 Moderating Role of MA

The study's findings provide empirical support for the negative moderating role of “metacognitive awareness (MA) in the relationship between emotional intelligence (EI) and creativity‐nurturing behavior (CNB).” While both EI and MA are essential for fostering teaching self‐efficacy, their interaction does not necessarily lead to a simple additive effect. Teachers with high levels of both EI and MA may prioritize emotional regulation and structured reflection (Ma and Liu 2024), potentially resulting in a diminished emphasis on creative instructional approaches. This aligns with the negative moderation hypothesis, which suggests that the presence of high MA reduces the intensity of the relationship between EI and CNB. The rationale behind this effect is that teachers with heightened MA might favor well‐structured, analytical teaching methods over spontaneous and creative strategies.

The findings indicate that at lower levels of MA, EI has a stronger effect on CNB, suggesting that creativity thrives when teachers are emotionally intelligent but not overly reflective about their instructional methods. However, as MA increases, the indirect effect of EI on CNB weakens, implying that excessive reflection and structured thinking could potentially constrain teachers’ ability to foster creativity. This underscores the importance of maintaining an optimal balance between EI and MA to effectively nurture creativity in the classroom.

### 7.7 Role of MA in the Indirect Relationship Between EI and TSE

The moderated mediation analysis further supports the notion that MA influences the indirect effect of EI on TSE through CNB. Prior research has established that MA is positively associated with teaching self‐efficacy (Alkan 2020; Yidana 2023; Yildiz and Akdag 2017). Teachers with higher MA are likely to engage in deliberate and strategic reflection on their instructional practices, which can enhance the effect of EI on CNB and, consequently, TSE. Conversely, when teachers exhibit lower levels of MA, they may be less reflective about the creative processes in their classrooms, leading to a weaker indirect effect.

The results of the study confirm that MA moderates this indirect relationship, with higher levels of MA reducing the mediating role of CNB between EI and TSE. Specifically, the effect of EI on CNB to TSE is more pronounced when MA is lower but becomes weak and nonsignificant when MA is high. This suggests that while MA is valuable for improving instructional strategies, excessive emphasis on structured reflection may inadvertently dampen the creative influence of EI. The slope analysis further reinforces this conclusion, illustrating that as MA increases, the ability of EI to positively influence TSE through CNB diminishes. This finding highlights the need for a balanced approach to fostering both EI and MA among teachers. Professional development programs should aim to cultivate EI while ensuring that metacognitive reflection does not hinder creative instructional practices. By striking the right balance, educators can maximize the positive effects of EI on self‐efficacy through CNBs, ultimately enhancing teaching effectiveness.

## 8 Conclusion

This study highlights the significant roles of EI, MA, and CNB in shaping Economics teachers’ teachers' self‐efficacy (TSE). The findings demonstrate that EI serves as a crucial factor in enhancing teachers’ confidence in their instructional abilities, particularly through its influence on CNB. Furthermore, the mediating role of CNB reinforces the idea that teachers who effectively nurture creativity in their students are more likely to experience greater self‐efficacy. However, the interplay between EI and MA presents a more intricate relationship, as higher levels of MA appear to moderate the effects of EI on CNB and, ultimately, TSE. This suggests that while reflective teaching practices are essential, an excessive focus on structured metacognition may diminish the creative aspects of teaching, thereby affecting the overall impact of EI on self‐efficacy.

The study's findings underscore the need for professional development initiatives that strike a balance between EI, metacognitive reflection, and creativity‐nurturing pedagogies. Institutions should prioritize training programs that cultivate teachers’ EI while fostering adaptive and flexible teaching strategies that incorporate both creativity and reflective practice. Additionally, policies that support creativity‐driven learning environments will be instrumental in strengthening Economics teachers’ self‐efficacy. By ensuring that teachers effectively integrate EI and MA in their instructional approaches, educational stakeholders can enhance the overall quality of teaching and learning, ultimately improving student outcomes in Economics education.

From a cognitive‐affective neuroscience and behavioral psychology perspective, the findings of this study shed light on the dynamic interaction between emotional regulation, cognitive monitoring, and professional functioning in educational contexts. EI, which involves processes such as emotional awareness, regulation, and interpersonal understanding, is underpinned by the coordinated activity of brain regions including the prefrontal cortex, amygdala, and anterior cingulate cortex. These systems facilitate not only affective processing but also adaptive behavioral responses essential for effective teaching. The study's finding that EI positively influences both CNB and teachers' self‐efficacy (TSE) underscores the role of emotional functioning in fostering proactive and confident instructional behavior.

MA, linked to executive control and higher‐order thinking, is associated with prefrontal cortex functioning and supports self‐regulation and reflective practice. However, its negative moderating effect on the relationship between EI and CNB suggests that excessive cognitive monitoring may interfere with the spontaneous and affect‐driven behaviors that support creativity. This highlights a complex interaction between affective and cognitive systems in shaping teaching behavior. The moderated mediation finding further reveals that MA can diminish the indirect impact of EI on TSE through CNB, emphasizing the need for a balanced integration of emotional and cognitive processes. These insights provide a valuable foundation for neuroscience‐informed teacher development programs. It is important to recognize that effective teaching relies on the integration of EI and metacognitive reflection, rather than an overreliance on either one. Educational stakeholders can therefore design interventions that strengthen teachers' emotional and cognitive adaptability. Such programs could enhance teachers’ brain‐aligned capacities to regulate emotions, support creativity in learners, and build professional confidence, ultimately improving behavioral outcomes in real‐world classroom settings.

## 9 Recommendations

The Ghana Education Service should integrate structured training programs on EI and MA into continuous professional development (CPD) initiatives for Economics teachers. These programs should focus on equipping teachers with strategies to regulate emotions effectively, reflect on their teaching practices, and nurture creativity in the classroom. Additionally, GES should implement mentorship programs where experienced educators can guide less experienced teachers in balancing structured reflective practices with creative instructional methods to enhance teachers' self‐efficacy (TSE).

Economics teachers should actively engage in professional learning communities and self‐directed professional development to refine their EI and metacognitive skills. They should adopt innovative teaching strategies that encourage creativity among students while maintaining structured reflective practices. Furthermore, teachers should embrace adaptive teaching approaches that allow them to balance their MA with CNBs, ensuring that their instructional methods remain dynamic and effective in fostering student engagement and learning.

The Ministry of Education should develop and implement policies that support a balanced integration of EI, MA, and CNB in the national curriculum for teacher training programs. Teacher education institutions should be encouraged to embed EI and metacognitive strategies into preservice training curricula to ensure that future educators are well‐prepared to foster creative learning environments. Additionally, the Ministry should provide funding and resources to support workshops, conferences, and research on best practices for improving teachers’ self‐efficacy through emotional and cognitive skill development.

Headteachers should create school environments that encourage both creativity and reflective teaching practices among Economics teachers. This can be achieved by organizing in‐school professional development sessions focused on EI, creative pedagogy, and metacognitive strategies. Furthermore, school leaders should foster a supportive culture where teachers are encouraged to experiment with new teaching methodologies and share best practices through peer collaboration. By providing an enabling environment, headteachers can enhance teachers’ confidence, creativity, and overall effectiveness in the classroom.

## 10 Contributions of the Study

This study makes significant theoretical, methodological, practical, and empirical contributions to the field of education, particularly within the context of Economics teachers’ instructional practices. Theoretically, the study advances the understanding of how EI and MA interact to influence teachers' self‐efficacy (TSE) through CNB. By integrating these constructs within a moderated mediation framework, the study extends existing theories on teacher self‐efficacy and creativity in education. Methodologically, the study contributes to the literature by employing a variance‐based structural equation modeling approach, which provides a robust statistical analysis of complex relationships. This methodological rigor enhances the validity of findings and offers a replicable analytical framework for future research exploring similar psychological and pedagogical constructs in education.

From a practical perspective, the study offers actionable insights for educators, policymakers, and educational institutions. The findings underscore the importance of balancing reflective teaching practices with CNBs to enhance teacher self‐efficacy, providing a foundation for targeted professional development programs. Empirically, this study enriches the body of knowledge on teacher education by providing context‐specific evidence from Ghanaian Economics teachers, highlighting how EI and metacognition shape instructional behaviors. This contributes to a growing discourse on teacher development in sub‐Saharan Africa, bridging gaps in existing research and offering insights applicable to similar educational settings globally.

## 11 Limitations

This study, while offering valuable insights into the interplay between “emotional intelligence, metacognitive awareness, creativity‐nurturing behavior, and teaching self‐efficacy among Economics teachers,” is not without limitations. First, the cross‐sectional design limits causal inferences, as relationships observed at a single point in time may not fully capture the dynamic nature of teacher development. Future studies could adopt longitudinal or experimental designs to establish causality more effectively. Additionally, the study relied on “self‐reported data,” which may be subject to social desirability bias; incorporating classroom observations or peer assessments could enhance the robustness of findings. Furthermore, the study was conducted within the Ghanaian educational context, and while the results provide valuable implications, generalizability to other educational systems may require further investigation. Future research could explore these relationships across diverse cultural and institutional contexts or examine the role of additional moderating variables, such as institutional support and curriculum flexibility, to provide a more comprehensive understanding of teacher effectiveness and professional development.

## Author Contributions

**Francis Arthur**: conceptualization, methodology, software, data curation, validation, visualization, investigation, formal analysis, writing–original draft, writing – review and editing. **Regina Okyere‐Dankwa**: writing – original draft, writing – review and editing. **Dominic Owusu**: writing – original draft, writing – review and editing. **Sharon Abam Nortey**: data curation, writing – original draft, writing – review and editing.

## Peer Review

The peer review history for this article is available at https://publons.com/publon/10.1002/brb3.70832

## Figures and Tables

**FIGURE A1:**
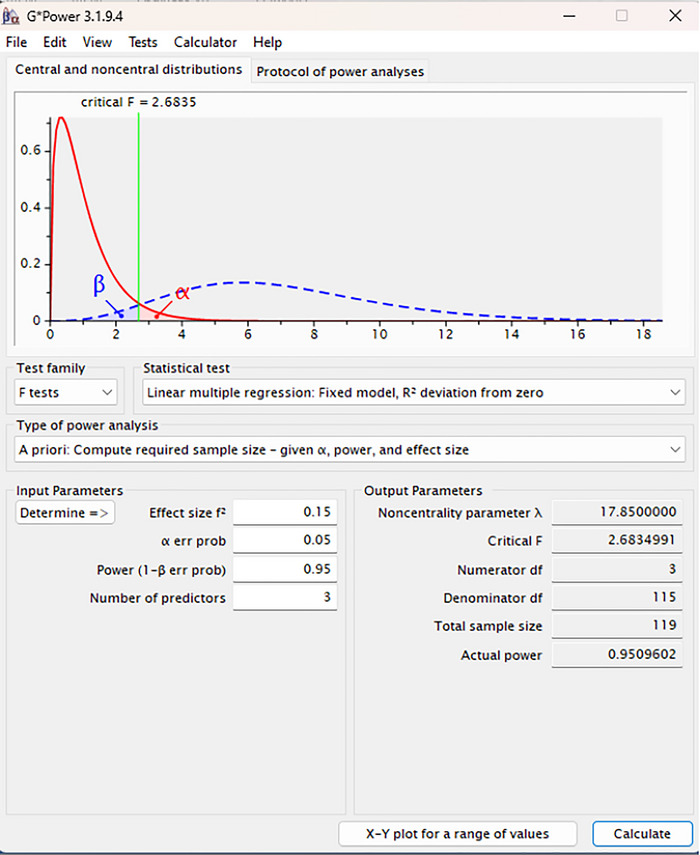
Sample size calculation from G*Power software.

**FIGURE B1:**
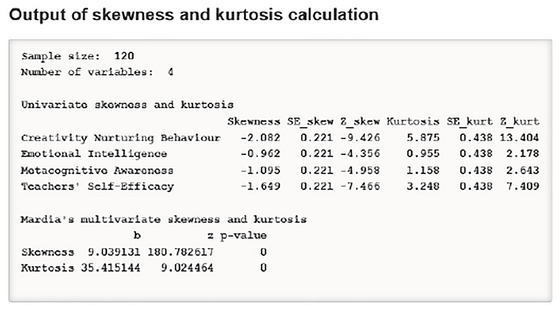
Univariate and multivariate skewness and kurtosis.

**FIGURE C1:**
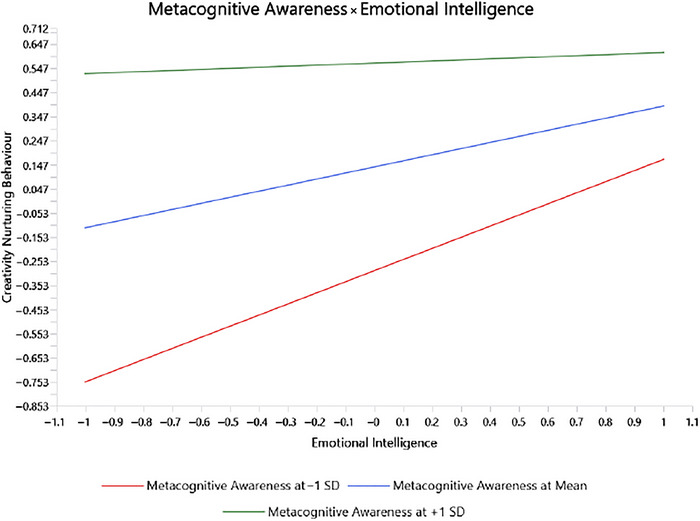
Moderated mediation slope results from SmartPLS 4.

## Data Availability

The data that support the findings of this study are available from the corresponding author upon reasonable request.

## Appendix A

### A.1

Figure A1

## Appendix B

### B.1

Figure B1

## Appendix C

### C.1

Figure C1
